# MOPSS: Toward high‐fidelity oligonucleotides for clinical applications

**DOI:** 10.1002/ctm2.809

**Published:** 2022-07-19

**Authors:** Hansol Choi, Sunghoon Kwon, Taehoon Ryu, Yeongjae Choi

**Affiliations:** ^1^ Department of Electrical and Computer Engineering Seoul National University Seoul Republic of Korea; ^2^ Bio‐MAX Institute Seoul National University Seoul Republic of Korea; ^3^ Interdisciplinary Program in Bioengineering Seoul National University Seoul Republic of Korea; ^4^ ATG Lifetech Inc. Seoul Republic of Korea; ^5^ School of Materials Science and Engineering Gwangju Institute of Science and Technology Gwangju Republic of Korea

1

Oligonucleotides (oligos) are nucleic acid chains designed and synthesized for various applications. Oligos can be used in different therapeutic functions, for example, antisense oligonucleotide (ASO), immune‐stimulatory oligo, aptamer, Cas9, miRNA, siRNA, and shRNA owing to their advantages of easy design and synthesis.^1^ Recently, the COVID‐19 pandemic has underscored the potential of oligo‐based technologies by promoting mRNA vaccines. Oligo‐based therapeutics have introduced a novel mechanism of directly silencing or enhancing gene function in vivo, which is different from the functioning of conventional drugs. The United States Food and Drug Administration (FDA) has approved several oligo‐based therapeutics, and more than 155 active clinical trials are currently ongoing, advancing the clinical use of oligos as a paradigm‐shifting methodology.^1^, ^2^

When utilizing oligos in clinical applications, fidelity is one of the most critical criteria affecting their performance.^3^ Oligos are synthesized by coupling oligo monomers individually, following the desired sequence. Errors in oligo sequences, including deletions, insertions, and substitutions, inevitably occur during synthesis, since the monomer coupling is not 100% efficient.^4^ For therapeutical uses, oligos are either hybridized to the target gene, genome, or RNA to regulate or edit gene expression (e.g., ASO, siRNA, sgRNA) or are translated in vivo to be utilized as a drug or immune stimulatory molecule (e.g., mRNA vaccine). Throughout the applications, even single‐base errors can lead to a dramatic decrease in efficiency or significant side effects. When oligos are hybridized to the target, the hybridization effectiveness can be reduced by a quarter if a single base error exists.^5^ As a result, the therapeutic performance may not be quantified with oligos that contain errors because the number and position of these errors are not predictable. In addition, if sgRNAs for gene editing (e.g., the CRISPR‐Cas9 system) have errors, the off‐target effect caused by incorrect hybridization can damage the functional genome. Furthermore, when oligos with synthetic faults are translated in vivo for mRNA vaccines, shortened or incorrect proteins may be produced in vivo, which may cause side effects.

To increase reliability and impact of oligo‐based therapeutics, it is necessary to ensure high fidelity. For this reason, we recently developed a highly parallel oligo purification technology (Multiplex Oligonucleotide Libraries Purification by Synthesis and Selection [MOPSS[) that isolates error‐free oligos from the ones with synthetic errors.6 MOPSS is a length‐based oligo purification technology. It is directed to the fact that most synthetic errors of oligonucleotides are length‐altered errors, such as deletions and insertions (indels). MOPSS measures the lengths of oligos in a highly parallel manner by coupling one nucleotide per cycle, followed by recognition of the nucleotide type (e.g., adenine, cytosine, guanine, and thymine), and selects a length‐matched population. For example, we could design oligos in which the nucleotide type 120 bp far from the bound primer is “adenine”; if the type is changed due to indels, the oligo might not be recognized by specific type of nucleotides (e.g., biotin‐modified thymine bases). In this case, only error‐free oligos were coupled with biotin‐modified dATPs, followed by selection using streptavidin magnetic beads. We demonstrated that this technology can be applied to oligo libraries consisting of more than 10 billion different sequences with distinct lengths in one pot. MOPSS is a meaningful discovery because it can prevent frameshift errors that occur from the translation of oligos with indels. In addition, MOPSS does not require any additional (dummy) sequences for purification and can be applied to oligos of various purposes, because the nucleotide‐recognizing region is located at a pre‐existing site (e.g., primer sequence). Therefore, we applied MOPSS to digital data encoded oligos, synthetic antibody encoding oligos, and human genome capture oligos from a previously reported article. In addition, we repurposed a next‐generation sequencing (NGS) instrument to an automated length measuring equipment for purification. We believe that MOPSS can be applied to the large‐scale production of oligos for clinical use due to its high scalability.

Even though MOPSS anticipates the achievement of high‐fidelity oligonucleotide therapeutics, further development of the technology is required. The main limitation of MOPSS is that it cannot eliminate substitution errors, because it is subject to a length‐based selection. When possible, it is desirable to eliminate substitution errors in oligos because these can be translated into wrong amino acids or a non‐synonymous mutation. Another limitation of MOPSS is the purification yield. We increased the ratio of length‐matched oligos from 83% to 97%; however, oligos with length errors were still present. MOPSS measures the length of oligos by coupling one nucleotide per cycle; although if a nucleotide fails to be coupled or more than one nucleotide is coupled, dephasing or pre‐phasing can occur. In this way, length measuring cycles are desynchronized and oligos with indels can be accidentally selected.

Non‐specific binding during the selection process, which relies on the biotin‐streptavidin interaction, might have contributed to the population of length‐altered oligos after purification. We believe that the aforementioned limitations of the technology can be further improved by engineering polymerases, optimizing enzymatic reaction conditions and time, and using more robust chemical interactions when selecting error‐free oligos. To apply these solutions, an in‐house apparatus rather than an NGS instrument is essential. Previously, we applied MOPSS to DNA; however, further development is also required to apply this technology to RNA because various clinical applications, including sgRNA and siRNA, require high‐quality RNA.

Error‐free oligos play an important role not only in clinical applications but also in synthetic biology, metabolic engineering, drug discovery, and biological studies.4 Recent efforts to synthesize oligos with lower error rates using an enzymatic synthesis approach have not shown significant improvement^7^; therefore, purification after synthesis is inevitable. A technology that can purify oligos by NGS analysis followed by single pick‐up^8^, dial‐out PCR,^9^, ^10^ and hybridization‐based purification^11^ has been proposed prior to our recent development.6 Although these technologies have shown potential, they have not yet been adopted by the industry due to their low scalability. We believe that MOPSS is suitable for the oligo‐therapeutics industry because of its potential to purify high complexity oligonucleotide libraries and its automated process. With oligos with increased fidelity, the clinical outcome of the pharmaceutical applications may be improved with lowered side effects and higher efficacy. An in‐depth study comparing the efficiency of applications following oligo's fidelity will be conducted in the future.

## References

[1] Kulkarni JA , Witzigmann D , Thomson SB , et al. The current landscape of nucleic acid therapeutics. Nat Nanotechnol. 2021;16(6):630‐643. 10.1038/s41565-021-00898-0 34059811

[2] Roberts TC , Langer R , Wood MJA . Advances in oligonucleotide drug delivery. Nat Rev Drug Discov.2020;19(10):673‐694. 10.1038/s41573-020-0075-7 32782413 PMC7419031

[3] It O , Kingdom U . QC in antisense oligo synthesis. Nat Biotechnol. 2002;20(9):871‐872. 10.1038/nbt0902-871b 12205500

[4] Kosuri S , Church GM . Large‐scale de novo DNA synthesis: technologies and applications. Nat Methods. 2014;11(5):499‐507. 10.1038/nmeth.2918 24781323 PMC7098426

[5] Relógio A , Schwager C , Richter A , Ansorge W , Valcárcel J . Optimization of oligonucleotide‐based DNA microarrays. Nucleic Acids Res. 2002;30(11):1‐10. 10.1093/nar/30.11.e51 12034852 PMC117213

[6] Choi H , Choi Y , Choi J , et al. Purification of multiplex oligonucleotide libraries by synthesis and selection. Nat Biotechnol. 2022;40(1):47‐53. 10.1038/s41587-021-00988-3 34326548

[7] Palluk S , Arlow DH , De Rond T , et al. De novo DNA synthesis using polymerasenucleotide conjugates. Nat Biotechnol. 2018;36(7):645‐650. 10.1038/nbt.4173 29912208

[8] Lee H , Kim H , Kim S , et al. A high‐throughput optomechanical retrieval method for sequence‐verified clonal DNA from the NGS platform. Nat Commun. 2015;6. 10.1038/ncomms7073 PMC4327316 25641679

[9] Schwartz JJ , Lee C , Shendure J . Accurate gene synthesis with tag‐directed retrieval of sequence‐verified DNA molecules. Nat Methods. 2012;9(9):913‐915. 10.1038/nmeth.2137 22886093 PMC3433648

[10] Lim H , Cho N , Ahn J , et al. Highly selective retrieval of accurate DNA utilizing a pool of in situ‐replicated DNA from multiple next‐generation sequencing platforms. Nucleic Acids Res. 2018;46(7). 10.1093/nar/gky016 PMC6283416 29361040

[11] Pinto A , Chen SX , Zhang DY . Simultaneous and stoichiometric purification of hundreds of oligonucleotides. Nat Commun. 2018;9(1):1‐9. 10.1038/s41467-018-04870-w 29941961 PMC6018234

